# Synopsis of *Plazia* Ruiz & Pav. (Onoserideae, Asteraceae), including a new species from northern Peru

**DOI:** 10.3897/phytokeys.34.6151

**Published:** 2014-01-31

**Authors:** Michael O. Dillon, Federico Luebert

**Affiliations:** 1 Botany Department, The Field Museum, 1400 South Lake Shore Drive, Chicago, IL 60605, USA; 2 Freie Universität Berlin, Institut für Biologie – Botanik, Altensteinstr. 6, D – 14195, Berlin, Germany and Departamento de Silvicultura y Conservación de la Naturaleza, Universidad de Chile, Santiago, Chile; 3 Present address: Universität Bonn, Nees – Institut für Biodiversität der Pflanzen, Meckenheimer Allee 170, D – 53115 Bonn, Germany

**Keywords:** Asteraceae, Mutisioideae, Onoserideae, *Plazia*, endemics, new species, flora of Peru, Department of La Libertad

## Abstract

A synopsis of *Plazia* Ruiz & Pav. (Onoserideae, Asteraceae) is presented, including the description of a new species, *Plazia robinsonii* M.O.Dillon & Sagást., from a locality c. 20 kms west of Huamachuco, Department of La Libertad in northern Peru. It most closely resembles *Plazia conferta* Ruiz & Pav., a narrow endemic from central Peru some 450 km to the south; however, the latter species has larger leaves and smaller capitula. *Plazia* is a small genus of four species confined to the Andean Cordillera of Peru, Bolivia, Chile, and Argentina. A distribution map of the four species, an illustration of the new species, a photograph of the holotype, and a key to species are provided.

## Abstract (es)

Se presenta una sinopsis del género *Plazia* Ruiz & Pav. (Onoserideae, Asteraceae), incluyendo la descripción de una nueva specie, *Plazia robinsonii* M.O.Dillon & Sagást., proveniente de una localidad c. 20 km al oeste de Huamachuco, Departamento de La Libertad, norte de Perú. Esta especie es similar a *Plazia conferta* Ruiz & Pav., un endemismo del centro de Perú, unos 450 km hacia el sur; sin emabrgo, esta última especie tiene hojas más grandes y capítulos más pequeños. *Plazia* es un género compuesto por cuatro especies restringidas a la Cordillera de los Andes de Perú, Bolivia, Chile y Argentina. Se inlcuye un mapa de distribución de las cuatro especies, una ilustración de la nueva especie, una fotografía del holotipo y una clave para las especies.

## Introduction

*Plazia* Ruiz & Pav. (Mutisioideae, Asteraceae) is a distinctive genus confined to the Andean Cordillera of Peru, Bolivia, Chile, and Argentina. It is easily recognized by its suffrutescent and decidedly woody habit with stems to a meter or more. The sessile leaves are tightly clustered and confined to the terminal 10–20 cm portions of the branch apices, the radiate capitula have ray florets with whitish to pink corollas and dark purple anthers long-exerted from the disc whitish florets.

Phylogenetic studies have shown that the genus *Plazia* belongs to the tribe Onoserideae, along with the genera *Aphyllocladus* Wedd., *Gypothamnium* Phil., *Lycoseris* Cass., *Onoseris* Willd., and *Urmenetea* Phil. (Panero and Funk 2008, Katinas et al. 2008, Luebert et al. 2009, Panero 2009). These studies also show that the genus *Plazia* forms a clade within the Onoserideae together with *Aphyllocladus* and *Gypothamnium*. Recently, Panero and Freire (2013) suggested the inclusion of the genus *Paquirea* Panero and S.E. Freire in the tribe Onoserideae, associating it with *Plazia*, but provided no phylogenetic evidence to support that.

**Figure 1.**
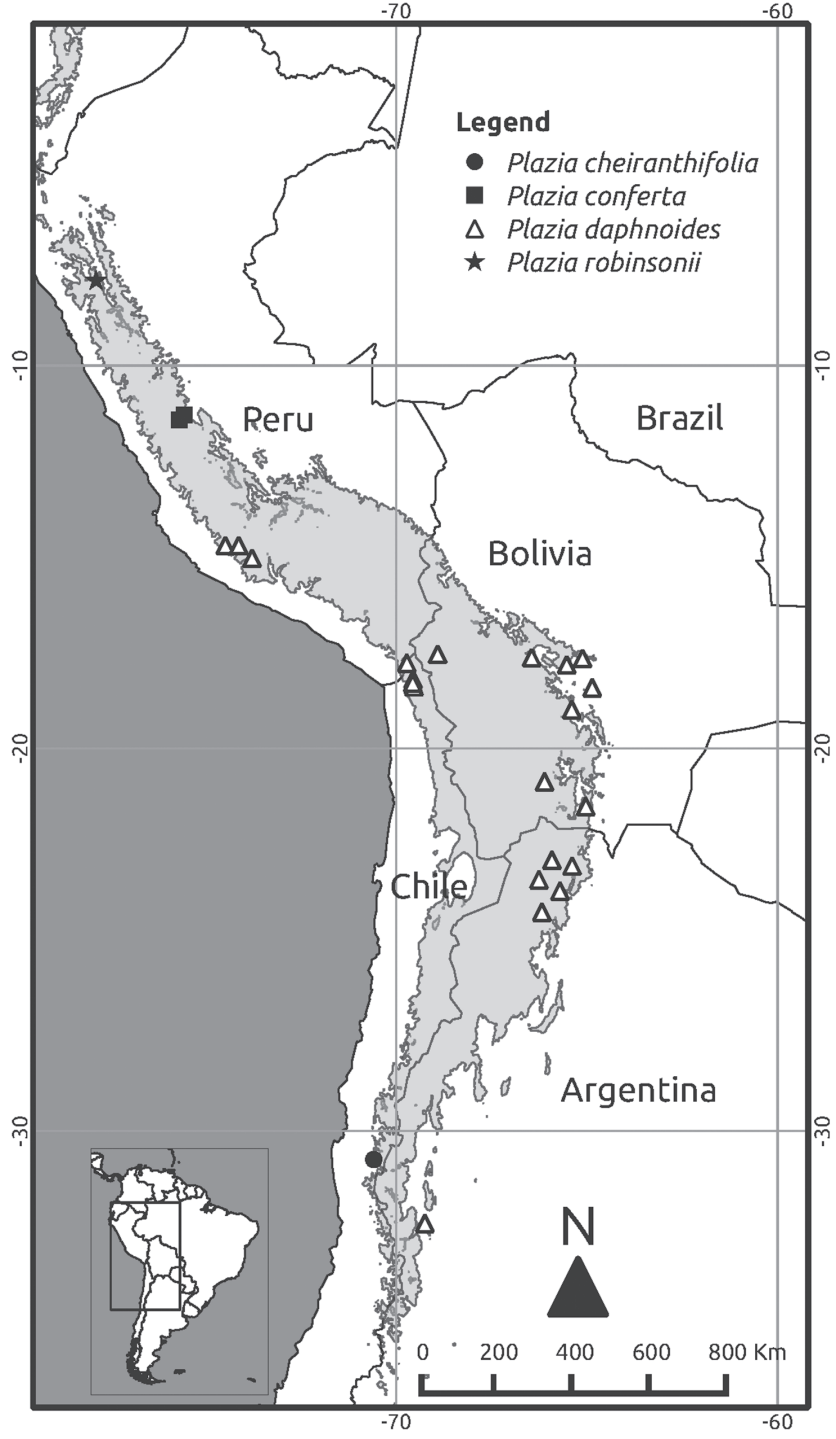
Distribution map of the currently recognized species of *Plazia*. The shaded area indicates high-elevation areas of the Andes above 3000 m. Locality data were obtained from Cabrera (1951), Ferreyra (1995), Missouri Botanical Garden (http://www.tropicos.org/), Instituto de Botánica Darwinion (through http://www.gbif.org/) and the herbaria ASU, B, F, HUT, P, SGO, US.

The distribution of these genera involves the Atacama Desert, the high Andes of central and northern Chile, southern Peru, northwestern Argentina and western Bolivia, the Chaco and Monte Regions, as well as the inter-Andean valleys of central and northern Peru (Cabrera 1951, 1977, Ferreyra 1980, 1995, Moreira-Muñoz and Muñoz-Schick 2007, Luebert et al. 2009). *Plazia* is restricted to the high Andes between northern Peru and central Chile and Argentina (Fig. 1).

## Taxonomy

### Plazia

Ruiz & Pav., Fl. Peruv. Prodr.: 92. 1794.

http://species-id.net/wiki/Plazia

Aglaodendron J.Rémy, Ann. Sci. Nat., ser. 3, 12: 175. 1849. Type: *Aglaodendron cheiranthifolium* J.Rémy = *Plazia cheiranthifolia* (J.Rémy) Wedd.
Harthamnus H.Rob., Phytologia 45(6): 451. 1980. Type: *Harthamnus boliviensis* H. Rob. = *Plazia daphnoides* Wedd.

#### Type.

*Plazia conferta* Ruiz & Pav.

#### Description.

Shrubs 1–2 m tall, the branches erect to ascending; stems lacking spines. Leaves simple, sessile, in whorls at branch tips; blades lanceolate to oblanceolate, glabrous to glandular, usually appressed, the margins entire. Capitulescences of solitary, terminal heads, sessile. Capitula heterogamous, radiate or more rarely homogamous, discoid; involucres cylindrical to campanulate; receptacles plane, glabrous; phyllaries 5–7-seriate, lanceolate; ray florets 5–25, the corollas ligulate-bilabiate, the outer lip 4-nerved, tridentate, the inner lip bifid; styles cylindric, glabrous, bifid, the branches short, inconspicuous; disc florets 7–40, the corollas tubular, actinomorphic, glabrous, the limb deeply 5-lobed, the lobes greater than 1/3 the length of the corolla, coiled; anthers linear, the terminal appendages lanceolate, fused into a column, truncate, the bases caudate; styles claviform, the branches short, rounded. Achenes glabrous or glandular-pubescent; pappus of scabrid bristles, isomorphic, yellowish. Chromosome number: unknown.

#### Distribution.

All species are confined to the Andean Cordillera and associated inter-Andean valleys (Fig. 1). Two species are confinded to Peru; both are rare and only known from a few collection localities. Another species is restricted to the Andes of central Chile. *Plazia daphanoides* Wedd. is the only wideranging species, being recorded from southern Peru and adjacent Argentina, Bolivia and Chile, usually in high-elevation, dry sites (Cabrera 1978, Ferreyra 1980, 1995).

#### Discussion.

The genus *Plazia* was described by Ruiz López and Pavón (1794) and they subsequently published its first species, *Plazia conferta* Ruiz & Pav. (1798). All efforts at locating material corresponding to their type collection from Peru have not been successful (Cabrera 1960).

Weddell (1855) provided an emended generic description for *Plazia* and he recognized three species: *Plazia conferta* Ruiz & Pav., *Plazia cheiranthifolia* (J. Remy) Wedd., and *Plazia daphnoides* Wedd. Reiche (1905) picked up the implied transfers by Hoffmann (1890–1894) and treated *Plazia* in Chile as having three species: *Plazia cheiranthifolia*, *Plazia pinifolia* (Phil.) O.Hoffm. [= *Gypothamnium pinifolium* Phil.], and *Plazia virgata* (Phil.) O.Hoffmann [=*Aphyllocladus denticulatus* (J.Rémy ex Gay) Cabrera]. Recent floristic treatments have accepted *Plazia* as distinct from *Aphyllocladus* and *Gypothamnium* (e.g., Cabrera 1978, Ferreyra 1995, Hind 2009, Marticorena and Quezada 1985, Zuloaga and Morrone 1999). The addition of the northern Peruvian species described here brings the total number of recognized species to four.

#### Key to species of *Plazia*

**Table:** 

1	Leaves oblong or oblong-spathulate, 8–18 mm long, 2–3.5 mm wide; capitula with 5–8(–10) ray florets, (7–)10–11(–12) disc florets; involucres cylindrical (Argentina, Bolivia, Chile, Peru)	*Plazia daphnoides* Wedd.
–	Leaves elliptic to oblanceolate or oblong, 22–42 mm long, 3–6 mm wide; capitula lacking obvious ray florets or more commonly with 15–25 ray florets, (9–)20–25(–40) disc florets; involucres campanulate 2
2	Leaves oblong, 28–30 mm long, 7.5–8 mm; corollas more or less isomorphic, obvious ligules lacking (Central Chile)	*Plazia cheiranthifolia* (J.Rémy) Wedd.
–	Leaves oblanceolate, 10–25 mm long, 1–3 mm wide; corollas dimorphic, outer florets with ligules obvious (Peru)	3
3	Involucre 25–30 mm in diameter; ray florets 15–20, disc florets c. 40	*Plazia robinsonii* M.O.Dillon & Sagást.
–	Involucre 12–16 mm in diameter; ray florets18–20, disc florets 10–20	*Plazia conferta* Ruiz & Pav.

### 1. Plazia cheiranthifolia

(J.Rémy) Wedd., Chlor. Andina 1: 12. 1855.

http://species-id.net/wiki/Plazia_cheiranthifolia

Aglaeodendron cheiranthifolium J.Rémy, Ann. Sci. Nat., ser. 3, 12: 175. 1849.

#### Type.

CHILE, Región IV, Coquimbo, *C. Gay s.n*. (holotype: P00703596!; isotype: P00703598!).

#### Description.

Shrubs to 1.5 m tall; stems very resinous. Leaves oblong, 28–30 mm long, 7.5–8 mm wide, sessile, apically acute, 3–5-nerved, entire, thickened, glabrous. Capitulescences of solitary terminal heads. Capitula solitary, sessile; involucres campanulate, 25–30 mm long, c. 25 mm wide; phyllaries 3–4-seriate; inner lanceolate, 25–28 mm long, c. 3 mm wide; florets numerous, isomorphic, the outer with corollas subligulate. c. 10, lobes strongly coilled, inner florets 20–25; all corollas lobes c. 7 mm long, c. 4 mm wide, the tube c. 13 mm long; pappus to 16 mm long. Achenes 6–7-ribbed, glabrous, linear oblong, c. 7.5 mm long, c. 1.2 mm wide.

#### Distribution.

While it was once considered extinct, this species was recently rediscovered in the Precordillera of Ovalle (Faúndez and Saldivia 2008) and the description provided here is largely derived from the specimens and data in that publication. Since this species appears to be restricted to an area of no more than 2 km^2^, it would be designated as “critically endangered” (IUCN 2001).

#### Discussion.

*Plazia cheiranthifolia* is a rare shruby species apparently confined to the central Chilean region near Coquimbo. It has the longest leaves of any species of *Plazia* and large capitula with broadly campanulate involucres. Rémy described as his *Aglaodendron chieranthifolium* as homogamous, all with bilabiate corollas with lobes of varying lengths. From the photo in Faúndez and Saldivia (2008), it appears there are perhaps nine subligulate corollas.

*Aglaeodendron cheiranthifolium* J.Rémy was based upon a collection by Claudo Gay deposited in Paris (P00703596). The isotype, also in Paris (P00703598), contains a small piece of paper c. 1 cm square, with the number “176” afixed to the lower left hand corner. This number corresponds to the page number of the collection citation in Rémy (1849). It should not be considered as an accession number for Claudo Gay as has been published in internet sources.

#### Specimens examined.

CHILE. Region IV. Prov. Limarí. Bocatoma Central Los Molles, Río Molles, 2590 m, 18 Jan 2007, *P. Saldivia et al. s.n*. (SGO154422!).

### 2. Plazia conferta

Ruiz & Pav., Syst. Veg. Fl. Peruv. Chil. 187. 1798.

http://species-id.net/wiki/Plazia_conferta

#### Type.

PERU. Junín: Acobamba [near Tarma], *H. Ruiz López & J.A. Pavón s.n.* (holotype: MA, n.v.).

#### Description.

Shrubs, branched, branches glabrous. Leaves oblanceolate, 22–42 mm long, 5–6 mm wide, glabrous, sessile, acute-mucronate, margin entire. Capitula with involucres 18–22 mm high, 12–16 mm wide; phyllaries 6–7-seriate, glabrous, lanceolate, the inner 22–25 mm long, 3–3.5 mm wide, acute, the outer gradually smaller; ray florets 18–20, the corollas 26–28 mm long, the tube 10–11 mm long, glabrous, the outer lip 14–15 mm long, 5–6 mm wide, 4-nerved, tridentate, the inner lip bipartite; disc florets 40–42, the corollas 15–18 mm long, the tube glabrous, the lobes 9–10 mm long, 1–1.2 mm wide, coiled; anthers 6–7 mm long. Achenes [ray] 4–5 mm long, 1.5–1.6 mm wide, glabrous; pappus c. 12 mm long; [disc] 4.5–5 mm long, 1–1.2 mm wide; pappus c. 14 mm long.

#### Distribution.

Endemic to an inter-Andean valley in central Peru from near Tarma; c. 3000 m. Given that this species appears confined to a single locality and of a few individuals, it would be considered “critically endangered” (IUCN 2001).

#### Discussion.

*Plazia conferta* is a rare species, and type material has not been located. No new material had been collected since Ruiz López and Pavón’s original gathering until a second collection was made by Felix Woytkowski at the type locality nearly 180 years after its original description. Cabrera (1960) was unsuccessful in locating Ruiz López and Pavón’s type material in the major European herbaria, including Madrid. We made inquiries to the Real Jardín Botánico in Madrid, but no collection of *Plazia* has surfaced as yet. Ferreyra (1980) cited duplicates of *Woytkowski 52* as occurring at MO and F, but after exhaustive searching, no duplicate collections were located, and subsequently the duplicates were not cited in Ferreyra’s Flora of Peru treatment (1995). Further, during this study, we were unsuccessful in our efforts to examine the Woytkowski collection at USM, and the description provided by Ferreyra (1995) was used to quantify the differences between that species and the new one described here.

There is an error in the citation of the generic description in Florae Peruvianae Chilensis Prodromus (1794), where page “104” is cited in Systema Vegetabilium Florae Peruvianae et Chilensis (1798) incorrectly, and the generic description is actually on page 92.

### 3. Plazia daphnoides

Wedd., Chlor. Andina 1: 13. 1855.

http://species-id.net/wiki/Plazia_daphnoides

Plazia daphnoides α *villosa* Wedd., Chlor. Andina 1: 13. 1855. Type. CHILE. XV Region, Prov. Parinacota: Cordillera de Tacora, entre Tacna et La Paz, *H. Weddell s.n*. (lectotype desiganted by Ferreyra 1995, pg. 83: P, P00703594!; isolectotype: ex P, F971331!).
Plazia daphnoides ß *glabrescens* Wedd., Chlor. Andina 1: 13. 1855. Type. BOLIVIA. Tomas Frias: Potosi, *A. d’Oribigny 1386* (lectotype, chosen here: P00793599!; isolectotypes: BR0000552180!, GH, P00703595!).
Harthamnus boliviensis H. Rob., Phytologia 45(6): 451. 1980. Type. BOLIVIA. Cochabamba: S E of Cochabamba, vicinity of Rodeo, 3500 m, 5 Mar 1979, *J. A. Hart 1739* (holotype: US2854177!; isotype: A, n.v.; photograph ex US, FM neg. 1944785!).

#### Description.

Shrubs to 2 m, resinous. Leaves sessile; blades oblanceolate to lanceolate, 10–18(–20) mm long, (2–)3–4 mm wide, apically acute, basally cuneate, glandular-pubescent, the margins ciliate. Capitula with cylindrical involucres (15–)18–20 mm high, 7–8(–14) mm wide; phyllaries 3–6-seriate, lanceolate, the inner 20–21 mm long, 2.4–3 mm wide, the outer smaller; ray florets (5–)7–8(–10), the corollas 20–21 mm long, the tube 11–11.5 mm long, the outer lip 9–9.5 mm long, 2–3 mm wide, tridentate, the inner lip bipartite, 6–7 mm long; disc florets (7–)10–11(–12), the corollas white, 13–14 mm long, the tube 10–11 mm long, the lobes 7–8 mm long; anthers 5–6 mm long. Achenes [ray] 4.5–5 mm long, 1.2–1.4 mm wide, glandular; pappus c. 13 mm long; [disc] 5–5.5 mm long, 1 mm wide, glandular; pappus c. 12.5 mm long.

#### Distribution.

In *Plazia*, *Plazia daphnoides* displays the widest distribution with collections from the Andean Cordillera of southern Peru, Bolivia, Chile, and Argentina (3000–4000 m). While locally it may come while locally may come under stress from habitat reduction, it would be considered as of “least concern” (IUCN 2001).

#### Discussion.

This species is distinctive with the narrowest capitula with the fewest ray and disc florets within the genus. Ferreyra (1995) lists the type specimen as collected by H. Weddell near the locality of Tacora, which is now in northern Chile.

Weddell (1855) failed to describe a nominative variety (p. 13); however, the manner in which he presented the material examined in his studies suggests that his first variety represented his nominative variety [α *villosa*, foliis utrinque villosis]. Examination of a large suite of collections has failed to find consistant morphological variation combined with any geographic pattern to support recognition of varieties in this taxon.

Robinson’s (1980) new genus and species, *Harthamnus boliviensis*, was discovered by him to be a synonym of *Plazia daphnoides* shortly after its publication.

#### Specimens examined.

ARGENTINA. Jujuy: Dept. Humahusca, Mina Aguilar, Espinosa del Diablo, 3800 m, 12 Jan 1968, *A.L. Cabrera et al. 18985* (LP, n.v.; SI014474!). Dept. Tumbaya, El Angosto de San José del Chañi, 3550 m, 26 Feb 1972, *A.L. Cabrera, J. Frangi, A.M. de Frangi, R. Kiesling & E.M. Zardini 22463* (LP, n.v.; P04318222!). Salta: Dept. Poma, Colres, 31 Jan 1944, *A. L. Cabrera 8331* (LP, n.v.; F1549306!, P02405527!).

BOLIVIA. Cochabamba: Sivingani, 11,000 ft, 24 Mar 1950, *W.M.A. Brooke 6219* (F1547725!). Tarija: Cercado, Tarija, 3900 m, 20 Feb 1904, *K. Fiebrig 3101* (F520491!).

CHILE. Region XV. Prov. Parinacota. Putre, 3500 m, 29 Dec 1995, *L. Landrum & S. Landrum 8883* (ASU0061610!); Quebrada Murmuntani, 3800 m, 13 May 2008, *M.A. Trivelli s.n*. (SGO156326!); Cordillera de Chapiquiña, 3600 m, 7 Mar 1927, *C. Troll 3235* (B!).

PERU. Ayacucho, 83 km W of Puquio, 76 km E of Nazca, 3430 m, 22 Jun 1978, A. *Gentry, M. Dillon, P. Berry, & J. Aronson 23273* (F1918361!).

### 4. Plazia robinsonii

M.O. Dillon & Sagást. sp. nov.

urn:lsid:ipni.org:names:77135762-1

http://species-id.net/wiki/Plazia_robinsonii

Figs 2 , 3

#### Type.

PERU. La Libertad: Prov. Huamachuco, Pallar – Huaguil, carretera a Tayabamba, 3000 m, 23 Jun 1974, *A. López M. & A. Sagástegui A. 8123* (holotype: HUT12930!, isotypes: F1863606!, US3266111!).

#### Diagnosis.

Plaziae confertae affinis, a qua foliis minoribus, capitulis majoribus et radiis 15–20 differt.

#### Description.

Shrubs to 1 m, the branches erect; stems lacking spines. Leaves simple, sessile, in whorls at branch tips; blades oblanceolate, 10–25 mm long, 1–3 mm wide, uninerved, adaxial and abaxial surfaces glabrous; margins entire. Capitulescences of solitary, terminal heads, sessile. Capitula heterogamous, radiate; involucres broadly campanulate, c. 25 mm wide, 30 mm in diameter; receptacles plane, glabrous; phyllaries 4–5-seriate, the outer ovate, 8–12 mm long, 4–6 mm wide, apically acuminate, the inner oblong 20–25 mm long, 4–5 mm wide, apically acute, ciliolate; ray florets 15–20, the corollas ligulate-bilabiate, whitish, the tube 4–5 mm long, the outer lip 4-nerved, 8–12 mm long, tridentate, the inner lip bifid; styles cylindric, glabrous, bifid, the branches short, inconspicuous; disc florets whitish, c. 40, the corollas tubular, actinomorphic, glabrous, the limb deeply 5-lobed, the lobes 5–8 mm long, c. 1/2 the length of the corolla, coiled; anthers linear, the terminal appendages lanceolate, fused into a column, truncate, the bases caudate; styles claviform, the branches short, rounded. Achenes [ray and disc] glabrous, 5-ribbed, 4–5 mm long; pappus of scabrid bristles, c. 15 mm long, isomorphic, pale yellow.

**Figure 2.**
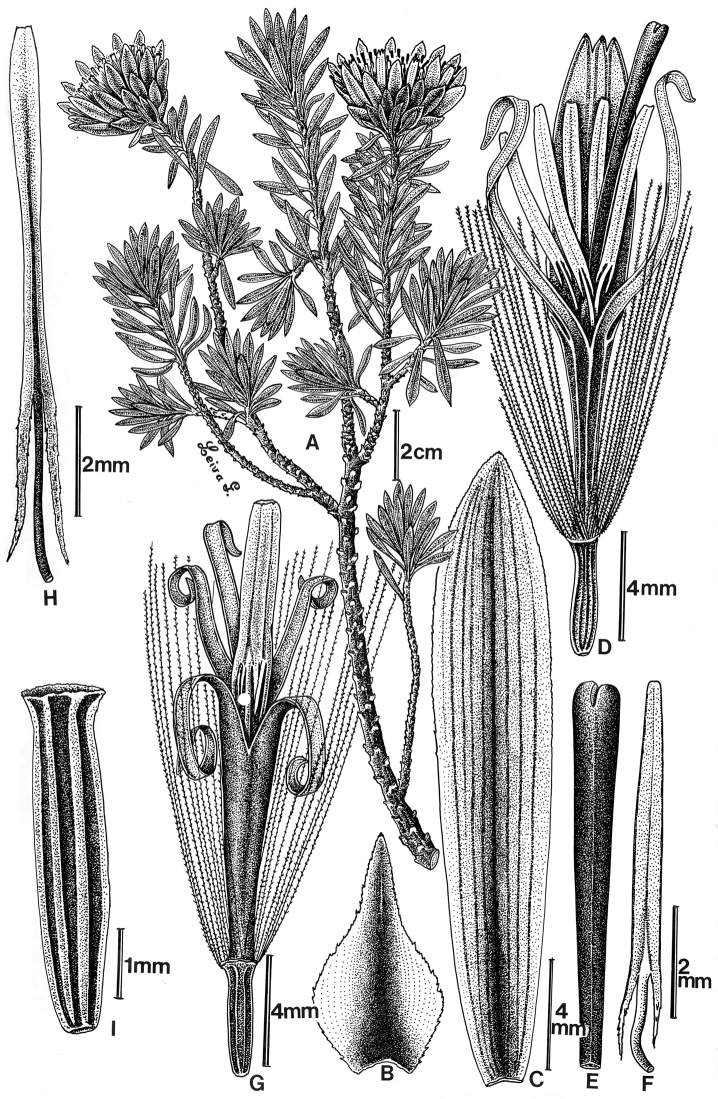
*Plazia robinsonii* M.O. Dillon & Sagást. (drawn from *A. López M. & A. Sagástegui A. 8123*). **A** Flowering branch **B** External phyllary **C** Internal phyllary **D** Ligulate floret **E** Terminal portion of the style of ligulate florets **F** Stamen from ligulate floret **G** Disc floret **H** Stamen from disc floret **I** Achene.

**Figure 3.**
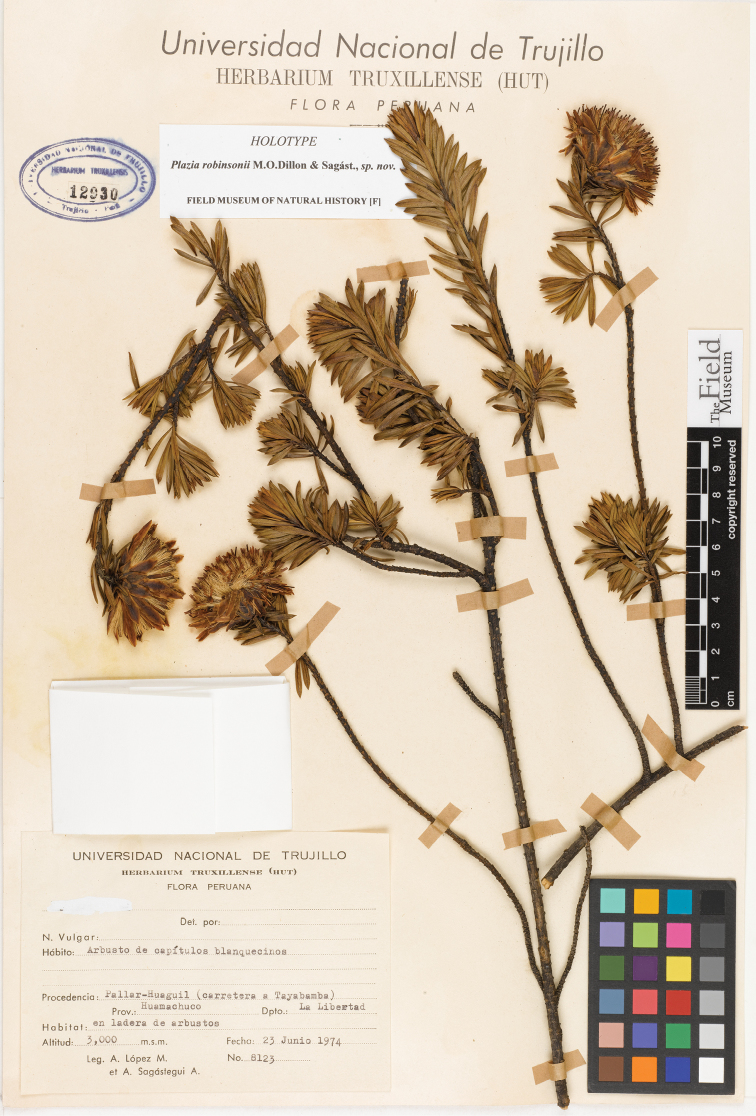
*Plazia robinsonii* M.O.Dillon & Sagást. Photograph of the holotype collection of *A. López M. & A. Sagástegui A. 8123* (HUT).

#### Distribution and conservation.

Known only from the type locality in an inter-Andean valley at around 3000 m (7°47.22'S, 77°52.27'W). The entire area surrounding Huamachuco and the road east to Tayabamba has been intensely cultivated for many years and is now highly disturbed by human pressure with expanding cultivation. Only fragmented small pockets of original habitats remain, usually in steeper quebradas. This species has not been recollected for 40 years and should be considered “critically endangered” (IUCN 2001).

#### Discussion.

The duplicates of the type collection of this new species were originally distributed under the generic name *Diplostephium* Kunth (1818), a member of the tribe Astereae. The overall morphology of the material does superficially resemble some members of *Diplostephium*, however, the bilabiate corollas, truncate style branches, and anther tails are typical for the Mutisieae not the Astereae. The new species was uncovered after the Flora of Peru treatment (Ferreyra 1995) was published and set aside for investigation. Only recently was the material encountered during routine filing, having been misplaced for nearly two decades.

The type locality is approximately 450 km north of its nearest congener, *Plazia conferta*, from near Tarma. Although the region where the original collections were made has been visited by numerous botanists, to our knowledge this species has not been recollected since 1974 when it was encountered by Abundio Sagástegui Alva and Arnaldo López Miranda. Casual efforts to find the plant again have not met with success. Given that the plant is quite showy and distinctive, it should not go undetected for long if it is indeed extant.

#### Etymology.

This species honors Dr Harold Robinson, Senior Research Curator at the National Herbarium, Smithsonian Institution. He suggested with his annotation of the US sheet, designated as an isotype, that this taxon was perhaps a new species of *Plazia*, and not an unusual *Diplostephium*, as had been suggested on the original label.

### Excluded names in *Plazia*

*Plazia acaciifolia* J.Koster = *Hyalis lancifolia* Baker

*Plazia argentea* (D.Don) Kuntze = *Hyalis argentea* D. Don ex Hook. & Arn.

*Plazia decussata* Hieron. (unpubl. herbarium name) = *Aphyllocladus decussata* Hieron.

*Plazia ephedroides* Hieron. (unpubl. herbarium name) = *Aphyllocladus ephedroides* Cabrera

*Plazia lorentzii* Hieron. = *Hyalis lancifolia* Baker

*Plazia pinnifolia* (Phil.) O.Hoffm. = *Gypothamnia pinnifolium* Phil.

*Plazia spartioides* (Wedd.) Kunth = *Aphyllocladus spartioides* Wedd.

*Plazia virgata* (Phil.) O.Hoffm. = *Aphyllocladus denticulatus* (J.Rémy ex Gay) Cabrera

## Supplementary Material

XML Treatment for Plazia

XML Treatment for Plazia cheiranthifolia

XML Treatment for Plazia conferta

XML Treatment for Plazia daphnoides

XML Treatment for Plazia robinsonii
